# The role of endothelial shear stress on haemodynamics, inflammation, coagulation and glycocalyx during sepsis

**DOI:** 10.1111/jcmm.15895

**Published:** 2020-09-19

**Authors:** Florea Lupu, Gary Kinasewitz, Kenneth Dormer

**Affiliations:** ^1^ Cardiovascular Biology Research Program Oklahoma Medical Research Foundation Oklahoma City OK USA; ^2^ Vasoactiv Biomedical Technologies Tulsa OK USA

**Keywords:** barrier function, coagulopathy, glycocalyx, haemodynamics, inflammation, mechanotransduction, sepsis, shear stress

## Abstract

Sepsis is a multifactorial syndrome primarily determined by the host response to an invading pathogen. It is common, with over 48 million cases worldwide in 2017, and often lethal. The sequence of events in sepsis begins with the damage of endothelium within the microvasculature, as a consequence of the inflammatory and coagulopathic responses to the pathogen that can progress to multiple organ failure and death. Most therapeutic interventions target the inflammation and coagulation pathways that act as an auto‐amplified vicious cycle, which, if unchecked can be fatal. Normal blood flow and shear stress acting on a healthy endothelium and intact glycocalyx have anti‐inflammatory, anticoagulant and self‐repairing effects. During early stages of sepsis, the vascular endothelium and its glycocalyx become dysfunctional, yet they are essential components of resuscitation and recovery from sepsis. The effects of shear forces on sepsis‐induced endothelial dysfunction, including inflammation, coagulation, complement activation and microcirculatory breakdown are reviewed. It is suggested that early therapeutic strategies should prioritize on the restoration of shear forces and endothelial function and on the preservation of the endothelial‐glycocalyx barrier.

## 1 INTRODUCTION

Endothelial cells (EC) line the luminal side of the vasculature in all organs and are uniquely situated to respond to systemic threats, such as sepsis. Sometimes referred to as ‘blood poisoning’, sepsis is a life‐threatening organ dysfunction syndrome caused by excessive and dysregulated host responses to microbial infection.^1^ Sepsis induces severe impairment of endothelial functions including vasomotor regulation, barrier function, inflammation and coagulation^2^ that contribute to microcirculation pathology and organ failure.^3^ The normal endothelium is both a blood‐tissue barrier and an endocrine organ. In addition to regulating vascular relaxation and microcirculation, endothelium modulates extravasation of fluids, solutes, macromolecules, hormones, blood and inflammatory cells. Endothelium influences blood fluidity, platelet adhesion and/or aggregation, leucocyte activation, adhesion and transmigration, and the balance between coagulation and fibrinolysis. EC also have an important role in immune responses and inflammation.^4^ Sepsis begins with vascular and shear stress (SS) dysfunction, increasing oxidative stress and inflammation, glycocalyx shedding, breakdown of EC junctions with blood barrier loss, paracrine and/or autocrine disorder, enhanced leucocyte adhesion and extravasation, and the initiation of a pro‐coagulant and anti‐fibrinolytic state.^2^

The most physiologically important stress on blood vessel walls is the tangential frictional force of flowing blood. Normal SS levels from laminar flow have a powerful homeostatic effect through mechanotransduction mechanisms^5^ involving the glycocalyx.^6^ The EC regulate blood flow, so when pathogens damage glycocalyx and the EC become dysfunctional, cascades of inflammation and coagulation escalate, which if uncorrected, lead to death. These cascades are the current theragnostic focus in sepsis therapy. While most sepsis therapies have been focused on late events, little attention was given to sustaining endothelial cytoprotection. Benjamin Franklin's wisdom of ‘an ounce of prevention is worth a pound of cure’ is applicable for sepsis therapy. Mediators of EC dysfunction might be viable targets for novel sepsis therapies in sepsis.^7^, ^8^ Maintaining circulation with adequate SS on vascular walls could reduce inflammation and its sequelae, and improve the delivery of anti‐infection agents.^9^ The role of SS on EC viability in sepsis and its implications in early interventional therapy has not been reviewed previously. We will evaluate SS effects on dysfunctional EC and the haemodynamic, inflammatory, coagulopathy and barrier loss sequelae during sepsis. We hypothesize that restoration of blood flow and physiological SS can help recover EC functions, and reduce morbidity and mortality.

## 2 SHEAR STRESS, HAEMODYNAMICS AND MICROCIRCULATION

Shear forces are vital for the homeostasis of blood vessels, triggering responses that affect EC morphology, function and gene expression.^10^ The EC are regulated biochemically by hormones, cytokines, neurotransmitters and mechanically by linear and tangential forces. Laminar SS frictional forces have critical roles on: (a) proliferation, survival or apoptosis of EC; (b) vascular smooth muscle tone; (c) EC antithrombotic activity; (d) production of growth factors and cytokines; (e) adhesive interactions with leucocytes; (f) EC production of reactive oxygen species (ROS) intracellular second messengers; (g) differentiation of immature endothelial progenitor cells and embryonic stem cells; (h) augmentation of mitochondrial ATP generation; and (i) regulation of over 600 EC genes.^6^, ^10^, ^11^, ^12^, ^13^, ^14^

**Figure 1:**
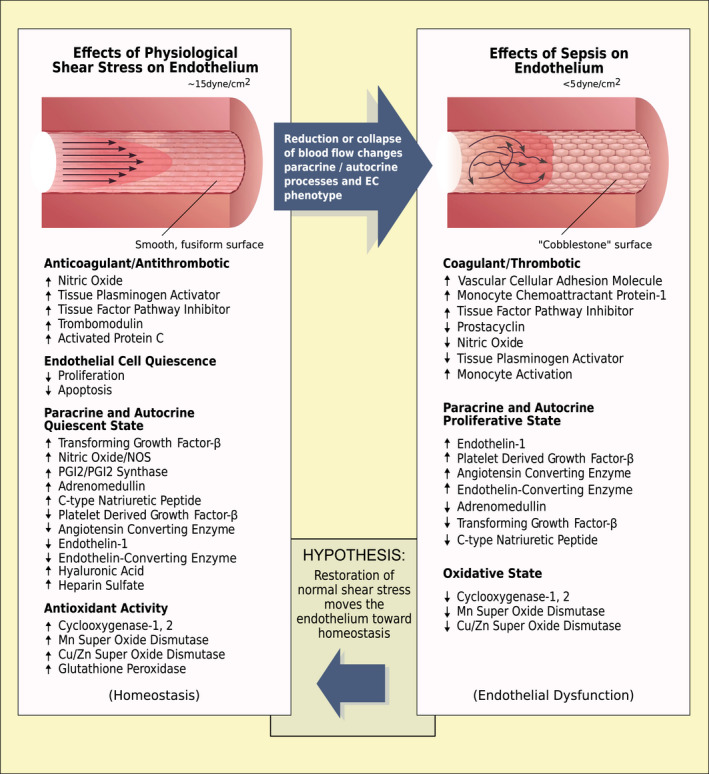
Effects of normal and reduced shear stress on the vessel wall. Left panel: normal endothelium receiving physiological (~15 dynes/cm^2^) shear stress from laminar blood flow patterns. The endothelium exhibits a quiescent fusiform smooth morphology with increased production of substances that are anticoagulant/antithrombotic and antioxidant. The endocrine status is summarized also, supporting the quiescent status. Right panel: dysfunctional endothelium resulting from low shear stress (<5 dynes/cm^2^). The endothelium displays a hypercoagulant/prothrombotic and pro‐oxidant state. Autocrine and paracrine changes listed, contribute to this dysfunctional vascular organ. In vitro and in vivo studies suggest the proposed hypothesis that restoration of laminar blood flow and adequate shear stress in sepsis would move the endothelium towards homeostasis

Effect of SS on EC is well understood in the context of atherogenesis. Normal SS is athero‐protective,^15^ while atherosclerosis develops at vascular sites of perturbed flow.^16^ Athero‐susceptible regions have pro‐proliferative and pro‐inflammatory gene expression, increased leucocyte adhesion and permeability, and decreased production of nitric oxide (NO). Whereas, laminar SS protects the function in health, inadequate SS during sepsis contributes to multiple pathologies. Circulatory collapse during sepsis severely impairs the SS mechanotransduction and initiates pathological changes. Figure 1 compares the normal homoeostatic role of laminar SS on EC with the dysfunctional effect of low flow and SS in sepsis. Signalling networks that impair the relationship between SS‐induced EC dysfunction and organ failure in sepsis have not been fully elucidated.

The conversion of SS into biological signals controlling endothelial functions is achieved by mechanotransduction.^17^ First sensed by the glycocalyx, SS is relayed to EC through complex signalling molecules that include integrins, tyrosine kinase receptors, G protein‐coupled receptors, ion channels and junction proteins. Other possible mechano‐sensors include caveolae and membrane lipid rafts.^18^ Mitochondria are involved with SS‐induced ATP generation and release, and Ca^2+^ signalling in EC.^13^ Redox signals to the EC exposed to non‐laminar SS or sudden changes in SS can initiate redox inflammatory responses that impair vascular health.^6^ Less is known about the effect of cyclic strain (tangential stress) on EC. While cyclic strain on pulmonary circulation during autonomous breathing was shown to maintain normal endothelial function,^19^ EC response to increased strain from positive pressure ventilation was shown to promote inflammation, leucocyte adhesion and vascular muscle contractility, leading to vascular dysfunction.^19^

Dysfunctional microcirculation during sepsis^20^ is multifarious, including abnormal leucocyte‐endothelium interactions, inflammatory and coagulopathic disorders, hemorheological defects (decreased red blood cell deformity, increased cell aggregation and viscosity) or functional shunting, all of which can be associated with abnormal SS on EC. Organ dysfunction or failure can be attributed to inadequate blood flow from generalized inflammatory and pro‐coagulant responses to infection.

Microcirculatory dysfunction is a key therapeutic target in sepsis and septic shock.^21^ Importantly, the loss of anticoagulation occurring during sepsis relates to reduced arterial pressure, blood flow velocity and SS. In the absence of physiological shearing forces, red blood cells, leucocytes and platelets more readily form stable aggregates with each other or EC lining vessel walls. This increases local blood viscosity and the probability of thrombosis at that site.^22^ The Protocolized Care in Early Septic Shock (ProCESS) clinical trial focused on improving the microcirculation.^23^ The trial has investigated alternative pressure and volume resuscitation strategies by administering fluids and vasopressors, while imaging the microcirculation. ProCESS showed that microcirculatory perfusion was not differentially influenced by pressure or volume resuscitations,^24^ but showed positive correlation between microvascular density at 72 hours and mortality, raising the prospect that early preservation of the EC function may be clinically significant. It was also proposed that NO administration might be a potent vasodilatory strategy. However, it is believed that NO^25^ is unlikely to work as it relaxes not only precapillary sphincters but also the arterioles, thus exacerbating pre‐existing sepsis hypotension. This leads us to the question: would early therapeutic strategies be more effective if they prevented the earliest events of sepsis, glycocalyx shedding,^26^ abnormal SS, and loss of EC homeostatic modulation of inflammation and coagulation?

## 3 SHEAR STRESS AND INFLAMMATION

Organ dysfunction during sepsis is caused by a multifactorial host response to infection. The immune response to invasive pathogens initiates a ‘cytokine storm’, where proinflammatory cytokines, such as tumour necrosis factor‐alpha (TNF‐α), interleukin (IL) 1β, IL‐6, IL‐12 and IL‐17 are among the most important.^4^ These cytokines trigger the expression of EC adhesion molecules and chemokines, such as monocyte chemotactic protein‐1 (MCP‐1), which recruit neutrophils and monocytes. These cells produce reactive oxygen and nitrogen species that may exacerbate EC and tissue injury.^27^ The consequent syndrome shows both excessive inflammation and immune suppression.^28^ The resulting pro‐inflammatory responses during sepsis involve the complement system, the coagulation cascade, vascular endothelium, neutrophils and platelets. Immune suppression results from reprograming of antigen‐presenting cells and apoptotic exhaustion of lymphocytes. As part of the homoeostatic regulation of the vasculature, SS plays a key role in regulating EC inflammatory responses.^29^ This was first elucidated for atherosclerosis, where the EC‐specific FOXP1 (fork head box P) transcription factor was identified as a gatekeeper of vascular inflammation by regulation of endothelial inflammasome components. FOXP1 is down‐regulated by Kruppel‐like‐factor2 (KFL‐2) and SS represses KFL‐2.^30^ This inflammatory mechanism also applies to sepsis.^31^ Normally shear forces induce an anti‐inflammatory state in EC through proteins and transcription factors that inhibit pro‐inflammatory signalling pathways. Leucocyte adhesion to the vessel wall is normally prevented by SS‐mediated downregulation of EC adhesion molecules and inflammatory proteins through suppression of inflammatory mitogen‐activated protein kinase (MAPK) and nuclear factor‐κB pathways. Inadequate low SS, as in sepsis, promotes inflammatory signalling. Hence, the inflammatory response elicited by infectious agents is exacerbated by the additional loss of the EC anti‐inflammatory protection when the glycocalyx is shed and EC become dysfunctional.

Sepsis‐induced inflammation may also be aggravated by the release of endothelial microparticles (EMP). Whereas normal SS inhibits EMP release, inadequate SS as well as other sepsis‐induced factors that injure EC (eg inflammatory cytokines, bacterial lipopolysaccharides, hypoxia and oxidative stress, coagulation enzymes and acute phase reactant proteins) can enhance EMP release.^32^ EMPs have pro‐inflammatory, pro‐coagulant and other pathological effects^31^ and can serve as a surrogate marker of endothelial function.^32^ Noteworthy in sepsis, low SS precedes the appearance of other generators of EMP.

## 4 SHEAR STRESS AND COAGULATION

Virchow published in 1856 his classical triad of factors that lead to thrombogenesis. The three components were abnormalities of blood vessel wall, blood constituents and blood flow. While Virchow's triad was initially described in relation to cancer‐related thrombosis, the concept is relevant to the pathophysiology of sepsis as the hypercoagulability, haemodynamic changes and endothelial dysfunction triad promotes sepsis coagulopathy.^33^

**Figure 2:**
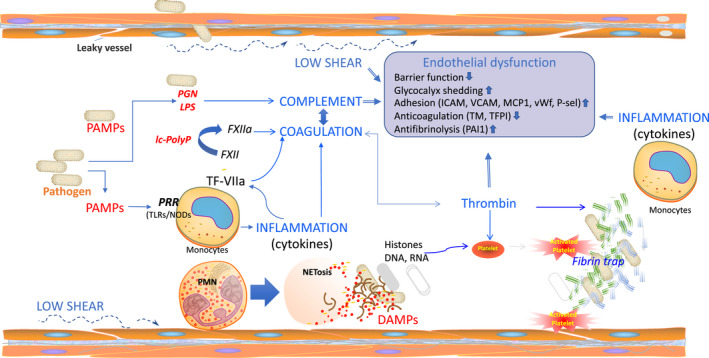
Crosstalk of haemodynamic forces, inflammation, complement and coagulation in the pathogenesis of sepsis coagulopathy. Early events are direct responses to the invading pathogens. Live bacteria or bacterial derived pathogen‐associated molecular patterns (PAMPS, such as lipopolysaccharide (LPS) or peptidoglycan (PGN) interact with pattern recognizing receptors (PRRs) inducing inflammatory responses. Cytokines like TNFα and IL1β are strong inducers of pro‐coagulant tissue factor leading to thrombin generation and platelet activation, then to fibrin deposition and microvascular thrombosis/DIC. Clotting factors and platelet consumption can lead to bleeding complications. Altogether these events can contribute to early organ failure and death. On the other side, pathogens PAMPs can trigger complement activation and C5a anaphylatoxin that is generated during this process is a potent leucocyte chemoatractant and activator. Activated neutrophils release their nuclear content as extracellular traps (NETs) rich in DNA and histones that contribute to pathogen clearance but are also potent platelet activators and are cytotoxic to the host cells. Histones and DNA can act as damage‐associated pattern molecules (DAMPs) that signal through the same sensor receptors PRR (TLRs/NODs) receptors leading to a further amplification of the inflammation, coagulation and complement activation. Moreover, neutrophil released myeloperoxidase contributes to the sepsis‐associated oxidative stress and organ failure. Altogether these events promote endothelial dysfunction characterized by decreased barrier function, increased glycocalyx shedding, increased leucocyte adhesion, decreased anticoagulation and fibrinolysis

The sequence of events leading to sepsis‐induced disseminated intravascular coagulation (DIC) is summarized in Figure 2. DIC results from the immune system's response to host invasion by microbial pathogens. Subsequent activation of clotting factors and platelets leads first to microvascular thrombosis then to bleeding, organ dysfunction, cell death and release of DAMPs that further amplify sepsis coagulopathy.^34^ Activation of coagulation by tissue factor (TF) is induced by pathogen‐associated molecular patterns (PAMPs) that signal through cell surface or intracellular receptors in monocytes. This process is further amplified by pro‐inflammatory cytokines induced by the same signalling machinery. TF triggers activation of the extrinsic coagulation cascade by binding and activation of Factor VII, resulting in activation of Factor X, thrombin generation and fibrin formation. Simultaneously, inflammation causes release of Platelet‐Activating Factor (PAF) that together with thrombin‐induced exocytosis of P‐selectin and von Willebrand factor (vWF) from EC increase TF expression on monocyte and platelet adhesion to large vWF strings anchored to leucocytes and endothelium, thus promoting microthrombosis.^35^ Haemodynamic forces regulate the function and the size of vWF. Thrombi formed under low or no SS (stasis) include many erythrocytes (red thrombi)^36^ while those formed under higher SS, such as in arteries are rich in platelets (white thrombi).^37^ Thrombogenesis is accelerated when the protease ADAMTS‐13 is consumed and cannot cleave the excessive amount of large vWF polymers.^36^ Microthrombosis leads to gradual consumptive thrombocytopenia, associated with increased mortality.^38^

Recently, it was demonstrated that activation of the intrinsic (contact) pathway can contribute to sepsis coagulopathy in response to certain pathogens, especially Gram‐positive bacteria, such as *Bacillus anthracis* ^39^ and *Staphylococcus aureus*.^40^ An important procoagulant stimulus is represented by the polyphosphates (poly‐P) released from bacterial pathogens or activated platelets, which promote Factor XII autoactivation,^41^ and platelet activation and consumption in the flowing blood.^42^ Contact activation serves as a nexus linking the main host defence systems: coagulation, inflammation and complement.^43^ Inhibition of contact activation with antibodies against factor XI decreased DIC, complement activation and inflammatory cytokines, and improved survival in bacterial sepsis.^44^

Another way to trigger activation of coagulation and exacerbate inflammation in sepsis is via damage‐associated molecular pattern (DAMP), such as, extracellular histones,^45^ nucleic acids^46^ and high mobility group box 1 (HMGB1)^47^ molecules that are released during cell death or shear‐induced neutrophil extracellular trap (NETs) release.^48^ Our group has shown that circulating histones are cytotoxic and thrombogenic, thus contributing to organ damage and DIC in sepsis.^45^ Circulating histones released during sepsis are degraded by plasma proteases, such as activated protein C^45^ and Factor VII activating protease (FSAP)^49^ or neutralized by negatively charged glycosaminoglycans associated with inter‐alpha inhibitor protein (IAIP).^50^

The inflammatory response simultaneously impairs multiple anticoagulant mechanisms involving EC and the glycocalyx mechanotransduction by SS. Tissue Factor Pathway Inhibitor (TFPI) is the main negative regulator of the extrinsic pathway by complexing with TF‐FVIIa and Xa.^51^ In addition, TFPI regulates coagulation through Protein S‐enhanced FXa inhibition and by decreasing the prothrombinase activity during the initiation phase of coagulation.^51^ TFPI expression and function are modulated by haemodynamic forces.^52^ TFPI is decreased in sepsis,^53^ due to degradation by proteolytic enzymes produced by the host, such as plasmin^54^ and neutrophil elastase^55^ or by bacterial omptins.^56^ Moreover, heparan sulphate (HS)‐bound TFPI can be shed from endothelial glycocalyx resulting in loss of anticoagulant activity of the EC surface.

Another key anticoagulant protein associated with the glycocalyx is antithrombin, a serpin‐type inhibitor of coagulation proteases. Antithrombin is consumed and functionally impaired when HS glycosaminoglycans (GAGs) are shed from the EC in sepsis.^57^

Thrombomodulin (TM), an integral membrane glycoprotein expressed on the luminal surface of vascular EC, is a key component of the TM‐Protein C (PC) system that controls the coagulation, inflammation and endothelial barrier functions. Thrombin bound on TM activates PC to activated PC (APC),^58^ a potent anticoagulant and anti‐inflammatory protein that cleaves two cofactors (Va and VIIIa) that are essential for the production of thrombin. During sepsis, both TM and PC are decreased due to lower synthesis and increased degradation. Moreover, the Endothelial Protein C Receptor (EPCR) and cofactor Protein S, which amplify the production of APC, are dysfunctional on damaged EC.^59^

Both cyclic strain and adequate SS collectively promote endothelial anticoagulation, cytoprotective and anti‐inflammatory homeostasis.^60^ Significant levels of TM are up‐regulated and released following 6‐12 hours of SS onset. With dysfunctional EC and low SS endothelial TM expression is functionally impaired. Administration of recombinant soluble TM reduces DIC in sepsis patients.^61^

Similar to coagulation, sepsis is a strong inducer of the complement system. Both the coagulation and complement systems are synergistic and cross‐regulated to protect the host.^62^ Complement activation is pro‐coagulant and coagulation proteases support complement activation. Anticoagulant pathways crosstalk with the inhibitory mechanisms of complement.^62^ The interactions between coagulation and complement are reflected also in haemodynamic forces on these systems. Mechanical forces such as SS counter the activation of coagulation and complement cascades, while inadequate flows promote blood clotting and complement activation. Physiological SS up‐regulates^63^ and pulsatile strain decreases^64^ the expression of clusterin, a complement inhibitory protein on EC. Physiological SS up‐regulates CD59, a major inhibitor of terminal complement complex formation, increasing endothelial resistance to complement‐mediated injury.^65^ Turbulent or oscillatory (non‐linear) flow induces EC expression of high amounts of properdin,^66^ a positive regulator of the alternative pathway of complement that is strongly induced during sepsis.

Complement and coagulation crosstalk also with NETosis, a recently described host defence mechanism involving released NETs, with extrusion of nucleic material rich in DNA, histones, enzymes and antibacterial peptides.^67^ NETosis occurs during bacterial sepsis, clearing pathogens from blood, preventing spreading to other tissues.^68^ Haemodynamics have a role in NET formation. Neutrophil crawling and trans‐endothelial migration are potentiated by adequate SS.^83^Arterial pressure gradients and high interstitial haemodynamic forces promote release of NET.^48^ As SS aids in recruitment of leucocytes at inflammatory sites, NETs promote coagulation by acting as scaffolds for thrombi formation.^69^ Similarly, NETosis synergistically promotes complement activation, while the complement activation products stimulate NETosis.

Summarizing, haemodynamic forces integrate three host defence systems—coagulation, complement and NETosis—^69^ that can contribute to immuno‐thrombosis,^70^ to locally immobilize, contain and kill microbial pathogens. When immuno‐thrombosis fails and bacteria spread in sepsis, uncontrolled activation of these three enzymatic cascades contributes to organ failure and death.

Therefore, coagulation, complement, NETs and haemodynamic pathways are important targets for sepsis treatment towards prevention of organ failure and death. First established as a potent inhibitor of sepsis DIC,^72^ APC was administered in a single successful clinical trial and became the only FDA approved drug (Xigris).^71^ It was removed from market when subsequent trials failed to confirm its initial promise.^72^ Currently, there is no drug specific for sepsis^73^; therefore, there is an acute need for novel therapies. Our group is investigating in preclinical models novel approaches targeting the intrinsic^44^ and common^74^ pathways of coagulation, as well as the activation of complement at C3^40^, ^75^ or C5^76^ levels. Today, therapy for sepsis focusses on antibiotics and haemodynamic support, including fluid resuscitation and vasoactive drugs aimed to correct hypotension and restore proper tissue perfusion.

## 5 SHEAR STRESS AND GLYCOCALYX BARRIER FUNCTION

**Figure 3:**
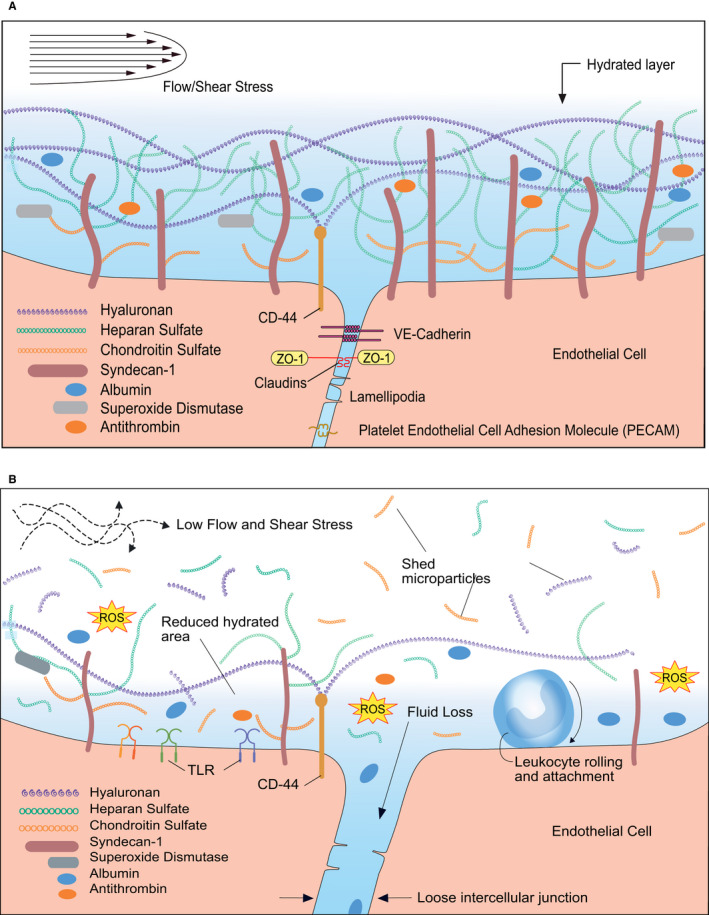
Barrier breakdown during sepsis. A, Organization of the glycocalyx coating the healthy endothelium exposed to adequate shear stress. A balance occurs between the synthesis of glycosaminoglycans (GAGs) and core proteins, and their enzymatic degradation. Lipid rafts carry glypican‐1 with its anchored heparan sulphate (HS) to the endothelial cell boundary (boundary clustering). Syndecan‐1 carrying HS and chondroitin sulphate (CS) along with caveolae containing localized glypican‐1 and HS do not move. Anchored caveolae are abundant on the endothelial apical surface and associate with glypican‐1. Shear stress induces ongoing synthesis of HS and CS and from their uniform distributions, numerous long stress fibres form and distribute in the apical parts of the endothelial cells where they stabilize caveolae and syndecans‐1. The endothelial actin cytoskeleton interacts with the transmembrane core protein syndecans‐1 and the caveolar structural protein caveolin‐1 for stabilization. The glycocalyx components HS, CS, glypican‐1 and syndecan‐1 are enriched on the apical surface, with nearly uniform spatial distributions. Intercellular junctions are stabilized by VE‐cadherin, claudins, lamellipodia and platelet endothelial cell adhesion molecules (PECAM). The hydrated layer of the normal glycocalyx allows for the containment of albumin, antithrombin and the antioxidant superoxide dismutase. This dynamic organization of the endothelial glycocalyx barrier enables mechanotransduction, modulates capillary (endothelial) permeability and is dependent on adequate shear stress from flowing blood. B, Changes in the organization of glycocalyx during early sepsis. PAMPs and inflammation products mediate the breakdown of the glycocalyx‐endothelial cell barrier (EG/EC). Subsequent hypotension and low shear rate reduce shear stress to non‐physiological levels results in shedding of the glycocalyx. The hyaluronan, HS and CS integrity is broken. The underlying EC becomes unprotected and mechanotransduction is lost, directly exposing EC to atypical shear stress, damaging the EC. Reactive oxygen species (ROS) gain access to the EC. Leucocytes have greater access to EC attachment and extravasation. Breakdown the intercellular junction from loss of adhesive molecules increases filtration, including loss of albumin into the interstitial space, increasing its osmotic pressure and exacerbating filtration and oedema. With this EG/EC dysfunction, perhaps most important is the inability of the EC to regenerate itself and at some point in sepsis, this dysfunctionality is irreversible

The foremost cause of organ failure in septic shock is the inability of microcirculatory delivery of sufficient oxygen and nutrients. Trans‐endothelial exchange is controlled by endothelial tight and adherent junctions, caveolae and the endothelial glycocalyx (EG). The junctions, composed of intercellular binding molecules located between the clefts of EC, include occludins and claudins for tight junctions, and cadherins and catenins for adherent junctions (Figure 3). The resulting size‐selective endothelial pores vary among tissues, being smaller in brain capillaries and larger in liver sinusoids.

Under healthy conditions, EG acts as a permeability barrier through its negatively charged sugar residues that function as a charged molecular sieve. Major constituents of the glycocalyx are proteoglycans (PG), including syndecans and glypicans that contain heparan sulphate (HS) and chondroitin sulphate (CS), and hyaluronans attached to EC via the cell membrane receptor CD44.^77^ The EG thickness varies with vessel diameter between 0.2 to 2 µm, depending on flow shear and organ‐specific vascular bed. Cell junctions, caveolae and glycocalyx play key roles in mechano‐sensing SS.

Hyaluronan synthesis is induced by laminar SS and reduced when EC are exposed to oscillatory (non‐laminar) blood flow.^26^ EC need hyaluronan to maintain their integrity and its synthesis is determined by EC metabolic status.^26^ Laminar SS controls hyaluronan synthesis via the shear‐responsive transcription factor KLF‐2,^78^ through a mechanism involving EC glycolysis.^26^ Adenoviral‐induced overexpression of KLF‐2 stabilizes the junctions and increases trans‐endothelial resistance in cultured EC,^79^ while loss of KLF‐2 contributes to EC barrier failure in inflamed or overventilated lungs^79^ and KLF‐2 up‐regulation can restore the barrier function. Thus, preservation or reconstitution of normal blood flow, SS and mechanotransduction may be viable strategies for conserving or restoring intact barrier function in inflammatory diseases.

During sepsis, normal Starling forces driving transcapillary exchange fail, with the breakdown of the endothelial barrier due to opening of EC junctions and EG degradation. EG loss is happening during early sepsis and is associated with increased morbidity and mortality in several sepsis‐related pathologies, such as acute respiratory distress syndrome (ARDS), trauma and ischaemia/reperfusion injury.^80^ At the onset of bacteremia, EG is exposed to various glycosidases, metalloproteases and enzymes released from activated neutrophils. Heparanase, the sole endoglycosidase that degrades HS^81^ is stimulated by inflammatory cytokines and reactive oxygen species.^82^ This enzyme is activated in sepsis, contributing to loss of cell surface HS that decreases the AT‐dependent anticoagulant function and predisposes to thrombosis.^82^

Hyaluronan metabolism is also affected by sepsis. Plasma levels of HA are strongly increased during sepsis and correlate with poor survival.^83^ Consequently, hyaluronan shedding has been suggested as a possible blood biomarker of early bacteremic sepsis.^84^ EG associated HA is cleaved by hyaluronidases and radical oxygen species into low molecular species that can sustain inflammation by signalling through TLRs.^85^

In addition to glycosidases, EG degradation during sepsis is mediated by multiple proteases that can cleave transmembrane proteins, including PG and release them into the circulation. Matrix metalloproteinases (MMP) superfamily members, including MMPs and disintegrin metalloproteases (ADAM), are major sheddases that cleave the ectodomains of transmembrane PGs such as syndecan‐1, the major endothelial HS‐PG. ADAM10 and ADAM1, alone or in combination are responsible for degradation of syndecans‐1 and 4, and of endomucin, a heavily O‐glycosilated EG sialomucin that protects the EC against inflammation.^86^ Furthermore, these enzymes can cleave the EC junction proteins VE‐cadherin^87^ and JAM‐A^88^ and activate TNFα.^89^ Another sheddase, ADAM15 was shown to induce vascular leakage during sepsis by cleaving CD44 and release of cell surface hyaluronan.^90^ Moreover, MMP‐9 produced by IL1β stimulated inflammatory cells was shown to induce shedding of EG by cleaving syndecan‐4.^91^

Glycocalyx shedding impairs SS mechanotransduction and signalling, contributing to EC dysfunction.^92^ It has been suggested that glypican‐1, a GPI‐anchored PG localized in lipid rafts and caveolae is the core protein that transmits the fluid share force sensed by the HS GAGs to the signalling machinery. Thus, glypican‐1 knockdown in EC completely blocked shear‐induced phoshorylation of eNOS and NO production.^93^ Similarly, heparinase treatment blocked NO production in response to SS^94^ further supporting the mechano‐sensing role of EG.

When the EC‐EG barrier becomes breached, capillary fluid flux greatly exceeds reabsorption, causing interstitial oedema and local ischaemia that accelerates tissue ischaemia.^84^

Endothelial junction proteins serve as cytoskeleton anchors for mechanotransduction of SS and control pore size and intercellular permeability.^95^ Maintenance and coordinated dynamic opening and closing of inter‐endothelial junctions are an EC homoeostatic function. Loss of the EG and opening of the inter‐endothelial junctions constitutes endothelial barrier breakdown and leads to excess filtration and hypovolaemia during sepsis. Endothelial barrier function is controlled by signalling via sphingosine 1 phosphate (S1P) and angiopoietins. S1P is carried in blood by albumin, high‐density lipoproteins and the erythrocytes, and it acts through S1P receptor 1 to enforce EC barrier function and to protect EG by inhibiting metalloproteases that degrade cell surface syndecan 1.^96^ The angiopoietin (Ang)‐Tie2 axis is another contributor to EG and barrier integrity that is affected by sepsis. Under normal conditions, Ang1 ligation to Tie2 receptor maintains vascular and EG integrity while Ang2 is a competing antagonist that has destabilizing effects on vascular integrity and stimulates EG degradation by stimulating the release of heparinase 1 from EC stores.^97^ Ang2 is stored in endothelial Weibel‐Palade bodies and released in response to secretagogues such as thrombin and histamine, abundantly available during sepsis. Ang2 levels are over 50‐fold increased during sepsis,^98^ and its amount is a strong predictor of mortality in critically ill patients.^99^ Tie2 activation was shown to provide endothelial protection by suppressing heparanase and promoting reconstitution of EG in human sepsis^100^

Beside its role in vascular permeability, EG has homoeostatic protective functions by regulating its anti‐inflammatory, antithrombotic and antiangiogenic properties. Branched GAGs are proposed sensors of SS acting as a mechanotransducer signalling platform to maintain multiple EC functions, including production of NO and EC adhesion molecules.^101^, ^102^ Important for sepsis recovery, adequate SS also induces repair and remodelling of the EG using sphingosine‐1‐phosphate and HS proteoglycans.^101^

As detailed above, EG is an important regulator of EC anticoagulant properties. HS proteoglycans are strong enhancers of antithrombin and can mediate TFPI binding on EC surface. Degradation of EG removes a frontline defender against pathogens. The sheddases responsible for EG degradation are induced by the same reactive oxygen species and pro‐inflammatory cytokines^103^ that induce activation of coagulation. Early degradation of the EG has multiple pathogeneses, including: (a) impaired bacterial clearance, (b) increased platelet aggregation and thrombosis; (c) deficient mechanotransduction, (d) increased vascular permeability due to loss of hyaluronan; (e) reduced EC growth and survival factors and antioxidant defence, (f) predisposition to apoptosis; and (g) shedding of microparticles that activate toll‐like receptors and exacerbate the innate immune response.

Of therapeutic interest are recent data that intravenous fluid resuscitation in sepsis failed to reduce mortality due to inducing shedding of the glycocalyx.^104^ Pre‐clinical models suggest crystalloid resuscitation degrades the EG through an unknown mechanism. Fluids are given to increase perfusion pressure by increasing cardiac venous return, pre‐load and stroke volume. Increased preload and atrial stretch cause release of atrial natriuretic peptide (ANP) that has been shown to degrade EG in animals and humans.^104^ Finally, it was shown in a subgroup of septic shock patients in the ProCESS clinical trial that sepsis severity and the volume of intravenous fluids administered for resuscitation were associated with EG degradation^104^ but causality was not proven.

In summary, sepsis syndrome includes two remarkable vicious cycles: the inflammation‐coagulation and the degradation of glycocalyx‐EC dysfunction cycles.^105^, ^106^ It appears that early therapeutic interventions could be aimed to normalizing blood flow and SS thus both optimizing the distribution of antimicrobials and preserving the integrity of the barrier functions.^107^ Therapeutic strategies proposed to prevent EG degradation and enhance restoration of the EG‐EC barrier^77^, ^108^ include: (a) heparin‐mimetic compounds; (b) S1P1 agonists; (c) heparinase inhibitors; (d) modulation of C5a, high mobility group box 1 and Tie‐2 receptor; (e) stimulation of Ang1 or inhibition of Ang2; (f) activation of Slit‐Robo4‐dependent signalling to enhance VE‐cadherin dependent junctions.^109^

## 6 DISCUSSION

### 6.1 Haemodynamics, blood flow and SS

Over 2500 years ago, Heraclitus of Ephesus postulated his *Panta rhei* (everything flows) as the very essence of life.^79^ One unifying therapeutic aspect of sepsis is early loss of microcirculatory homeostasis. Additionally, dysfunctional microcirculatory perfusion includes insufficient gas and nutrient delivery, decreased capillary density, and stopped or intermittent flow.^110^ Maintenance of endothelial function at the microcirculatory level is critically dependent upon haemodynamics and EG/EC mechanotransduction. Fundamentally important is the recognition of EG loss that begins in the inflammatory phase of sepsis.^111^ At some point in cytokine‐mediated breakdown of the EG/EC barrier, septic shock is irreversible. Perhaps, early therapy focused on preserving and maintaining the endothelium should be prioritized.^110^ For the homoeostatic processes of capillary diffusion, filtration and recruitment to take place in heterogenous tissues, it requires an intact, tissue‐specific, functioning endothelium with intact glycocalyx.^2^, ^112^ At the macrohemodynamic level, adequate perfusion pressure to provide downstream capillary hydrostatic pressure is necessary. However, systemic haemodynamics are weakly correlated with microcirculatory parameters. Normal microcirculatory flow and SS are necessary for homeostasis, including EC growth, maintenance and their vasomotor, paracrine and autocrine functions. Dysfunctional EC lose their homoeostatic autoregulation that is essential for the renal, cardiac and cerebral circulations. This perhaps explains the futility of administering fluids in late septic shock when the barrier is broken, leading to high capillary permeability.^2^ Subsequent interstitial oedema increases microvascular resistance, a positive feedback cycle that escalates septic shock. Shedding of EG also breaks down the plasma osmotic pressure at the luminal EC surface and increases filtration, adding to this vicious cycle. Monitoring the EC/EG status at the bedside remains a primary obstacle in evaluating the pathogenesis of EC dysfunction during sepsis.^2^

Presently, there is no standard clinical biomarker for sepsis, and none have been shown to correlate with reduced mortality. The most measured biomarkers are pro‐calcitonin, C‐reactive protein and blood lactate, which is an indicator of later stage tissue hypoperfusion and organ damage. Blood lactate measurement has low diagnostic sensitivity and specificity.^111^ Many sepsis biomarkers have been evaluated.^113^, ^114^, ^115^ Over 170 biomarkers (mostly prognostic) have been identified^116^ but because of important early detection of EC dysfunction, this review suggests those surrogates of EG breakdown receive greater attention.^2^ Proteases, soluble VCAMs, glycocalyx components and haemostatic proteins TF and PAI‐1, selectins and integrins are such early breakdown indicators. Sepsis is defined as a severe enough infection to cause organ dysfunction,^1^, ^114^ but the predecessor of organ dysfunction is EG/EC barrier breakdown. Hence, perhaps theragnostic biomarkers of EG shedding should be prioritized, including HS, sialic acid, hyaluronic acid and syndecan‐1.^103^ The EG begins degradation and shedding in the early inflammatory phase of sepsis where metalloproteinases, heparinase and hyaluronidase (activated by reactive oxygen species and pro‐inflammatory cytokines) cause shedding. Clinical studies have correlated the presence of EC/EG blood components with organ dysfunction, severity and mortality of sepsis.^103^ Over‐aggressive fluid therapy exacerbates EG degradation, when perhaps EG preservation should be a therapeutic goal.

### 6.2 Inflammation, coagulation and SS

Damage to the endothelium prevents it from functioning normally as a brake on the inflammatory and coagulopathic cascades that characterize sepsis. Pathogen‐induced release of inflammatory cytokines, such as IL‐1b and TNFα induce EC up‐regulation of leucocyte adhesion molecules, which contribute to intravascular leukostasis and further loss of SS. EMP levels rise, further enhancing inflammation. PAMPs also initiate complement activation. Complement is important for antimicrobial defence, but persistent complement activation can result in excess tissue damage and organ failure.

PAMP‐induced expression of TF on monocytes initiates activation of the extrinsic clotting cascade and thrombin generation, which leads to fibrin deposition, platelet consumption and intravascular thrombosis. The coagulopathy is also promoted by the endothelial loss of TM and EPCR, two EC membrane proteins that promote PC activation, as well as the loss of TFPI, an inhibitor of the extrinsic pathway of coagulation.

**Figure 4:**
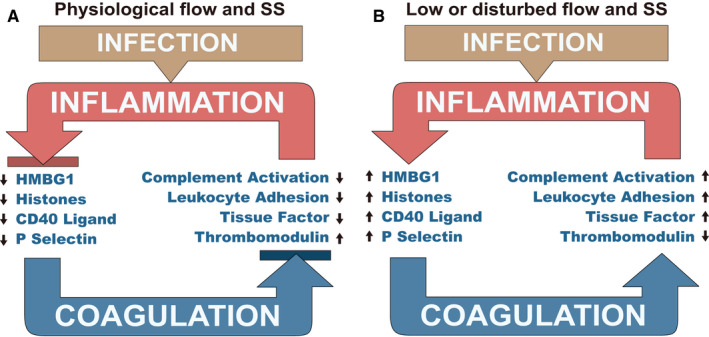
Autoamplification between infection, coagulation and inflammation. A, Homoeostatic EC inhibition of an underlying positive feedback cycle that is expressed during sepsis. In sepsis, inflammation promotes coagulation that further promotes inflammation. This cycle is sequestered normally and disabled by EG/EC barrier functions. The EC homoeostatically suppresses complement activation, leucocyte adhesion and tissue factor activity while maintaining thrombomodulin. The EC are anti‐inflammatory and show reduced HMBG‐1 or CD40 ligand‐mediated stimulation and no release of P‐selectin or histones. This positive feedback cycle in inhibited. B, Autoamplification during sepsis with dysfunctional EG/EC barrier. Infection either directly activates of the intrinsic pathway through activation of factor XII or induces tissue factor expression via toll‐like receptors and cytokine signalling. Coagulation leads to platelet activation, releasing CD40 ligand that amplifies inflammation, release of P‐selectin which aids in leucocyte trafficking, and with microcirculation ischaemia leads to the release of HMGB 1 or histones that further trigger inflammation and tissue damage. The resultant amplified inflammatory response leads to additional tissue factor formation, thrombomodulin down‐regulation, complement activation and leucocyte activation, further stimulating coagulation. Unchecked, this cycle leads to devastating inflammatory and coagulation‐mediated injury, multiple organ failure and death

During sepsis, inflammation, coagulation and complement activation are inextricably linked in a vicious cycle, where inflammation promotes coagulation that further begets inflammation^73^ (Figure 4). This cycle is promoted when sepsis is accompanied by low SS, raising the hypothesis that normalization of flow and EC SS would reduce both the inflammation and coagulation induced by sepsis. Clinical trials that sought to modulate the inflammation and coagulation cascades by inhibiting thrombin generation with heparin or endogenous anticoagulants have failed to improve patient outcomes.^73^ As SS effect on normal endothelium promotes anti‐inflammatory and anticoagulant functions, we hypothesize that insufficient SS and EC dysfunction in sepsis is amplifying the inflammation‐coagulation cycle, leading to multiple organ failure.

Morbidity in sepsis is not caused mainly by pathogen toxicity on the host but rather is the resultant of aberrant host responses,^1^, ^117^ illustrating a vicious cycle where the inflammation‐coagulation interplay leads to tissue hypoxia and organ failure.^118^ During sepsis, multiple events feed into this amplification loop, where glycocalyx is shedding, endothelium becomes dysfunctional, vessels become leaky, hypotension causes low shear rate and SS, while there is systemic inflammation, complement activation, increased leucocyte adhesion and hypercoagulation due to TF expression and imbalanced anticoagulants (thrombomodulin, AT and TFPI). (Figure 4B). Collectively, these effects are pro‐inflammatory, pro‐coagulant and support the release of DAMPs, which further incite positive feedback.

A clear correlation between haemodynamic forces and the inflammation‐coagulation axis was shown in the case of venous valves, where low or no shear (stasis) decreases shear activated transcription factors FOXC2 and Prox1 that control the expression of TM and EPCR, thus decreasing the anticoagulant potential while increasing leucocyte adhesion and inflammation.^119^ If in early sepsis, the ECs could remain functional with an intact glycocalyx, adequate SS and mechanotransduction, they would likely show down‐regulation of both pro‐coagulant and pro‐inflammatory effects, thus impeding the autoamplification cycle (Figure 4A). Likewise, if functional EC and adequate SS would be restored during sepsis, SS would drive antithrombotic and anti‐inflammatory phenotypes, and would help vascular healing by directing endothelial progenitor cells (EPC) to EC injury sites.^11^

### 6.3 Barrier breakdown and SS

Adequate shear rate and stress are necessary for maintaining vascular barrier homeostasis. Sepsis is a sequential syndrome beginning with pathogen‐induced systemic inflammation, initiating EG shedding followed by endothelial dysfunction. The EG’s proteoglycans and glycosaminoglycans normally attach circulating plasma proteins such as albumin, forming a protective EC surface layer, crucial for functioning and maintenance of the vascular barrier.^80^ As this protective mechanotransduction complex is shed, there begins loss of EC vasomotor, anti‐inflammatory and anticoagulant functions. Concomitantly, physiological SS is lost causing excess NO production, vasodilation, excessive capillary filtration, hypotension with resulting decreased flow. SS is an important vital haemodynamic force necessary for EC repair and blood vessel formation.^11^ Another positive feedback mechanism (autoamplification) is initiated when the EG/EC barrier becomes dysfunctional and its restorative‐rebuilding functions are lost. Furthermore, with EC/EG barrier breakdown and excessive filtration, there is increased leucocyte traffic, inflammation and interstitial hydrostatic pressure increases that closes off capillaries and impairs transcapillary exchange. As the EG degradation and shedding occur early in sepsis and is associated with increased morbidity and mortality, perhaps, both preservation and restoration of the EG should be therapeutic priorities in sepsis.^80^, ^103^, ^107^

## 7 SUMMARY

There are multiple definitions of sepsis/septic shock, which predicate their therapeutic approaches, but one unifying concept summarizes them all, an early loss of microcirculatory homeostasis. Haemodynamic homeostasis and its feedback and feedforward controllers are dysfunctional when tissues are not receiving enough flow, gases and nutrients during sepsis.^120^ Additionally, alterations in microcirculatory perfusion include decreased capillary density and capillaries with stopped or intermittent flow.^110^ Of the multiple feedback mechanisms failing during sepsis, a fundamental one appears to be maintenance of endothelial function at the microcirculatory level, which is dependent upon local haemodynamics and mechanotransduction imposed on the EC via the EG. This review points towards the importance of EG/EC loss that begins early in the inflammatory phase of sepsis and, if uncorrected leads to organ dysfunction and death.^111^

## CONFLICT OF INTEREST

The authors declare no competing financial interests.

## AUTHOR CONTRIBUTION

**Florea Lupu:** Conceptualization (lead); Investigation (lead); Visualization (lead); Writing‐original draft (lead); Writing‐review & editing (lead). **Gary Kinasewitz:** Investigation (equal); Writing‐original draft (supporting); Writing‐review & editing (equal). **Kenneth Dormer:** Conceptualization (lead); Investigation (lead); Visualization (lead); Writing‐original draft (lead); Writing‐review & editing (equal).

## Data Availability

Data sharing not applicable to this article as no datasets were generated or analysed during the current study.
